# Towards Non-Invasive Extraction and Determination of Blood Glucose Levels

**DOI:** 10.3390/bioengineering4040082

**Published:** 2017-09-27

**Authors:** Catherine Todd, Paola Salvetti, Katy Naylor, Mohammad Albatat

**Affiliations:** 1 Faculty of Computer Science and Engineering, Hawaii Pacific University, Honolulu, HI 96813, USA; 2 Ophthalmology, Moorfields Eye Hospital Dubai, Dubai Healthcare City 505054, UAE; paola.salvetti@moorfields.ae (P.S.); katy.naylor@moorfields.ae (K.N.); 3 Engineering and Information Sciences, University of Wollongong in Dubai, Knowledge Village 20183, UAE; mohammad.albatat@hotmail.com

**Keywords:** diabetic management, blood glucose measurement, non-invasive monitoring, intelligent decision support, acetone extraction

## Abstract

Diabetes is a condition where the body is incapable of proper utilization of glucose and one that, if not properly managed, can lead to critical illness. Glucose monitoring and decision support is vital in avoiding potential adverse health effects. Current methods mainly involve invasive blood extraction for the purposes of blood glucose level notification, yet such methods rely on active user participation and subjective interpretation of the result. This paper reviews existing research in methods of extraction and monitoring of glucose levels. The purpose of this paper is to examine blood glucose extraction methods in addition to indicators of blood glucose level, toward development of an innovative, non-invasive extraction technology. Decision support methods are also analyzed toward customized, automated, and intelligent diabetic management.

## 1. Introduction

Diabetes is a medical condition that occurs when the body is not able to appropriately utilize glucose as a form of energy. When glucose is at a normal level, it enables the body to operate effectively and provides a vital form of fuel for cell function [1]. High sugar levels in the bloodstream can cause complications by slowly damaging the cells in the pancreas and preventing them from producing insulin, a hormone that allows the body to utilize glucose found in food as a form of energy [1]. High blood glucose levels can internally damage the body, a condition known as atherosclerosis, and lead to critical health conditions such as hardening of the blood vessels [2]. Regular events of hyperglycemia (high blood glucose) can cause permanent organ damage [1]. If blood glucose is not properly regulated, it may rise to dangerous levels that can cause internal organ failure, kidney disease, stroke, heart attack, vision loss, a weakened immune system, poor blood circulation to the feet, and slower healing of wounds [1,2]. In effect, diabetics must constantly monitor their blood glucose [2]. This requires self-motivated efforts to avoid diabetes-related problems that have burdensome consequences on quality of life, including regular administration of medications and procedures required to address these complications.

There are two types of diabetes, each varying in cause of onset of the disease as well as complications and management strategies [3,4,5]. In type 1 diabetes mellitus, the body completely stops regulating blood sugar by not producing insulin. Type 1 diabetics must regulate their blood glucose manually through the external provision of insulin via injections, which must be issued daily for the recipient to survive [5]. This type of diabetes usually occurs at a young age but can also occur in older adults [5]. Type 2 diabetes occurs when the body is unable to produce sufficient insulin or is unable to process insulin properly. This form of diabetes is usually onset due to lifestyle and typically leads to fewer complications when compared to type 1 diabetes. Diabetes may develop irrespective of gender, age, genetics, or ethnicity [2], but the chance of being diagnosed with type 2 diabetes increases with age [5]. People who are over 40, are overweight, and have family members with a history of diabetes are more likely to develop type 2 diabetes, although it is becoming more prevalent in younger persons [5]. People who have family members or close relatives that are diagnosed with type 1 diabetes have an increased chance (1 in 7) of acquiring the disease than those that do not have the same genetic predisposition [4]. Diabetes may also be caused by other factors such as high blood pressure, physical inactivity, obesity, and high levels of cholesterol [5].

Diabetes-related deaths in 2012 numbered 1.5 million, while an additional 2.2 million deaths were due to high blood glucose [3]. The WHO projects that, in 2030, diabetes will be the seventh leading global cause of death [3]. In 2014, 422 million people had diabetes, representing 8.5% of the global population over the age of 18 years [3]. Given the high global incidence and potential adverse impacts of diabetes, in addition to global growth in diabetic-related deaths, there is a recognized need to provide ease of measurement of blood glucose levels in addition to lifestyle decision support toward proper management of the condition. 

This paper examines the two forms of diabetes, the physiological and chemical symptoms of the disease, and the evaluation of diabetic detection and treatment options, toward the design, development, and testing of a new electronic device with decision making support for diabetic management. The remainder of the paper examines the symptoms, treatment, and management options of the disease. Invasive and non-invasive approaches for diabetic monitoring are explored in the review of the research literature. Existing technologies for diabetic detection are examined, in addition to monitoring strategies, related treatment options, and decision support systems. The review is intended to identify state-of-the-art technologies that facilitate diabetic monitoring as well as research challenges in these areas. The strengths, contributions, and research deficits of the existing work are examined for the purpose of proposing a novel technique for non-invasive glucose monitoring and management of diabetes that combines a unique hardware and software solution.

## 2. Methods for Diabetic Management

### 2.1. Symptoms, Management, and Treatment

People who suffer from type 1 diabetes and type 2 diabetes will frequently experience physical symptoms that may be easily detected, including an increased hunger and/or thirst, unexpected weight loss, frequent urination, blurred vision, irritability, numbness or tingling in the extremities, frequent infections in the skin, gum or bladder, slow wound healing, or extreme and unexplained fatigue [6]. Such physiological symptoms of diabetes enable the disease to be self-monitored if not monitored by a care giver. Chemical symptoms of diabetes provide a consistent indication as to the status of the diabetic patient, including their blood glucose levels [6]. Physical symptoms may be readily detected through simplistic visual observation with the limitation of this being subjective and difficult to quantify. Chemical symptoms typically require a more invasive or analytical investigation [6], but they provide better quantification of diabetic status.

Those that suffer from type 1 diabetes or type 2 diabetes must engage in self-management strategies to retain acceptable levels of personal health and avoid irreversible damage to the body. Engagement in physical activity helps the body to process insulin better, enabling conversion of glucose into energy for cell growth, function, and regeneration [6]. Those diagnosed with type 1 diabetes and a subset of those with type 2 diabetes require administration of insulin injections [6]. A subgroup of people diagnosed with type 2 diabetes may manage the disease with oral medications rather than injections. These oral agents assist the pancreas in producing greater amounts of insulin and facilitate the body’s processing efficiency of the insulin as it is produced [6]. People diagnosed with diabetes currently must learn to regularly monitor their blood glucose to ensure it is well balanced and at an acceptable level to minimize future health-related complications.

Diabetic management requires personal use of a device that is capable of detecting blood glucose and displaying the reading [7,8,9]. A diabetic patient usually interprets the reading on their own accord. Blood glucose levels that provide an indication as to the status of diabetes are measured in mg/dL and mmol/L, with a reading range of 0 to 100 mmol/L inclusive [9]. Diabetics do not typically survive beyond a range of 700 mg/dL (39 mmol/L), with readings from devices not usually exceeding this threshold [9]. While extremely high readings are a cause for concern, low blood sugar readings are also dangerous. However, an interpretation of readings is somewhat subjective since this depends on lifestyle and current health status. Management will subsequently vary in terms of type of treatment required. Typically, a blood glucose reading between 0 and 85 mg/dL (0–4.6 mmol/L) may cause hypoglycemia and if immediate medical attention is not sought, this may lead to a comatose state [9]. Readings between 85 and 210 mg/dL (4.6–11.35 mmol/L) are typically considered within normal range [9]. Readings from 210 to 350 mg/dL (11.35–19 mmol/L) are considered as high [9]. Readings beyond this range are typically considered extremely high [9]. Depending on lifestyle, there are exceptions to preferred bands. High levels of activity, such as engagement in a sport or exercise, may require a higher reading than is typical, since if the reading is taken prior to physical exertion a higher reading enables the blood sugar to fall to a level that would be considered normal during the activity, ensuring that blood glucose does not fall too low during the state of exertion [9]. One should therefore anticipate upcoming activity or inactivity, for assessment of adequacy of the blood glucose level and subsequent treatment. 

### 2.2. Invasive Assistive Devices for Diabetic Monitoring and Their Use

To monitor blood glucose, diabetics must measure this level regularly, at least several times a day: after waking, before each meal, two hours after each meal, and prior to sleeping [8,9]. The frequency of measurement of glucose levels depends on the frequency of daily meal consumption. The most common method for the self-assessment of blood sugar levels is the use of a glucometer and test strips. Using the device, a finger is pricked to release a small sample of blood onto the test strip, which is then analyzed by the glucometer that outputs the blood glucose reading. Advances in glucometer device function has resulted in higher speeds of automated blood processing that provides a fast, easy mechanism for diabetics to monitor their levels. However, a high proportion of diabetics are reluctant to use self-administrative devices such as the glucometer, either skipping regular monitoring checks or abstaining entirely from the process. According to the American Diabetes Association, 21% of adults who are diagnosed with type 1 diabetes mellitus rarely check their blood sugar [10]. 

The psychological association between the results from the device and one’s identity may alter frequency of glucometer use [6,11]. While the device simply outputs a blood glucose reading, the quantitative measurement may also have an association of elation or depression depending on whether the reading satisfies expectations and may either motivate or demotivate the user for the degree of future device use [6]. The attached meaning that the person associates with the output reading may be related to the concept of testing, where the user is inferring a pass or fail grade based on the result. If this result is negative and is reiterated, it can demotivate the recipient from future device use [6].

Frequent, ongoing monitoring of a medical disorder can serve as a personal reminder of the disease, which may cause demotivation to use the glucometer [6,11]. Those that suffer from diabetes may wish to alleviate their preoccupation with it by avoiding such reminders. In addition, a person may feel as though the device is controlling their routines, with actions bound by the regularity and the anticipated result of the testing [6]. In the case of testing prior to a meal, checking blood glucose may inhibit one from eating the quantities or food types that they would otherwise choose to ingest. Throughout an array of lifestyle activities, the monitoring of blood glucose levels requires task interruption and a level of disconnection with current context [6]. Privacy during medical administration may also be a challenge if the user is in a public location [6]. Glucometers and their administration for the detection of blood glucose levels may therefore lead to one or more psychological demotivators that impede regular monitoring, which is reported in approximately 48% of diabetic cases [6]. These demotivators increase the risk of ineffective, improper, and infrequent diabetic management and treatment. 

Implantable glucose microchips offer reliable glucose measurement for detecting and quantifying changes in glucose levels. A wirelessly powered implantable electrochemical glucose sensor with RFID tag technology for data communication provides a small-scale, integrated chip solution for the determination of glucose levels, with high sensitivity [7]. While such devices offer direct and accurate measurement of blood glucose, patients may be psychologically opposed to receiving an implantable device as compared to non-implantable options. 

While the glucometer remains a popular and proven device of choice for blood glucose level determination, non-invasive strategies [9,12] seek to overcome direct blood extraction through analysis of other bodily fluids, while intelligent systems offer potential for decision support following glucose reading output to reduce demotivators associated with self-administration and self-management. The following section provides a comprehensive analysis of non-invasive techniques for blood glucose extraction and determination.

### 2.3. Non-Invasive Devices for Diabetic Monitoring

Spectroscopy involves the study of objects based on their wavelengths when they are emitting as well as absorbing light. Spectroscopic techniques have been used for non-invasive methods of blood glucose measurement, including near-infrared spectroscopy (NIR), Raman spectroscopy, bio-impedance spectroscopy (BIA), and thermal emission spectroscopy [9]. NIR uses infrared to capture reflected light from body tissue, indicating a level of blood glucose [9]. Raman spectroscopy exposes a subject to radiation where the light reflected during exposure is based on frequency shifting, indicating the level of blood glucose [9]. Health concerns relating to IR and radiation exposure are subject to contention. BIA applies principles of passive electrical properties of organic tissues, applying lower power excitation to liquids or tissues in the body, noting measurement changes as indicating blood glucose levels. The process requires patients to rest for one hour prior to measurement, which is inhibitive for daily, regular use. Thermal emission spectroscopy (TES) measures IR signs produced in the body as a result of glucose concentration changes [9].

Reverse iontophoresis refers to the passing of low level current passing the skin for the transport of both charged and neutral particles [12]. Two major transmission systems are included: electro-migration and electro-osmosis. Electro-migration is the movement of ions across the skin under the direct impact of an electric field [12]. Electron fluxes are changed into ionic fluxes by the electrode responses, and ionic transport continues through the skin to maintain electro-neutrality [12]. The aggregate charge transported relies upon the quality of the electric field and the duration of the application [12]. Iontophoresis sets in motion a few ions across the skin, which compete to convey a small amount of the current [12]. Shortcomings of transdermal glucose extraction through reverse iontophoresis include device function as that of an auxiliary to invasive measurement, notable lag and inconsistency of reading in comparison to results of the glucometer (direct blood measurement), long device calibration times, the production of skin irritations and blistering, and result variation as affected by environmental variables [12].

Sonophoresis transiently expands skin penetrability such that different medications can be conveyed non-intrusively and reveals potential for use in non-invasive glucose monitoring [12]. While this method proves workable for glucose measurement, it has only been tested in rats, where results have shown some degree of skin irritation [12]. Prototyping has not reached the stage of human subject testing. In addition, experimental error ranges are high, with a mean relative error of 15% [12].

Fluorescence-based methods for the determination of blood glucose levels are used to evaluate a variety of chemicals extracted from the human body during the measurement and classification process. These include folate, glucose, and retinal binding protein that provides vitamin A status [12]. Methods involving fluorescence, including electroenzymatic glucose oxidization, require the sample to be in contact with the sensor during chemical measurement, wherein fluid is directly extracted from the human body [12]. Fluorescence techniques would prove to be painless and reversible; while they show viability in measuring blood glucose, such processes have only been applied in vitro with cell culture models—not on human subjects [12].

Electromagnetism has been investigated for non-invasive glucose monitoring [12]. An electromagnetic sensor can determine the concentration of glucose through variation in the blood’s dielectric parameters—for example, its conductivity, as detected through changes in eddy current [12]. Changes in glucose concentrations therefore invoke conductivity fluctuations, as detected by the electromagnetic coils [12]. Electromagnetic techniques reveal a desired linear relationship between the modulus of output to input signal, and the glucose level. However, experiments have only been performed in vitro and not on human subjects. Further, a workable prototype has not yet been constructed, and results show variability from environmental interference.

## 3. The Proposed Method for Non-Invasive Glucose Extraction

### 3.1. Chemical Relationships between Acetone and Blood Glucose

In spite of the lack of reliable non-invasive techniques for diabetic blood glucose monitoring and lifestyle management decision support, there is evidence to suggest potential for non-invasively extracting and mapping acetone levels from human fluid to a blood glucose level, which may then be automatically and intelligently analyzed to support more appropriate lifestyle choices, such as regulating sugar intake. This section focuses on the chemical relationship between acetone, present in human breath, and blood glucose. The next section introduces intelligent decision support systems to show potential for customized algorithm development for diabetic management. Finally, this paper concludes with the proposal of a non-invasive device for extraction and classification of blood glucose levels based on acetone, as extracted from the human breath.

Ketosis is the process by which the body burns fat to make energy. During this process, Β-hydroxybutyrate (β-HB), commonly known as 3-hydroxybutyric acid, a ketone body, is created by the liver typically from oxidation of unsaturated fats, and is transferred to peripheral tissues for use as an energy source [13]. A ketone body essentially comprises three molecules: acetoacetate, β-HB, and acetone. β-HB and acetoacetate transport energy from the liver to alternate tissues and acetone is produced by unconstrained decarboxylation of acetoacetate [13]. Ordinarily, ketosis can demonstrate that lipid metabolism has been enacted and the pathway of lipid degradation is in place. Normal ketosis is prevalent in many circumstances, for example, during fasting, after delayed activity or after a high-fat eating routine [13]. Pathological reasons for ketosis include organ failure, diabetes, youth hypoglycemia, corticosteroid or growth hormone deficiency, intoxication with liquor or salicylates, and a few intrinsic mistakes of metabolism [13].

Diabetic ketoacidosis (DKA) is a dangerous internal condition that most commonly happens in those recently diagnosed with diabetes. This condition is a result of insufficient insulin production by the pancreas [8]. This condition can also develop in type 2 diabetics who have poor diabetic control and management [8]. Due to a lack of insulin in the bloodstream, a rise in blood sugar concentration occurs, which in turns prompts fat, sugar, acid unevenness, water, and protein build-up. Currently, a diagnostic test for DKA evaluates patient urine. However, the test is more sensitive to acetoacetic acid than acetone, rendering this test ineffective for the successful extraction of acetone for patients with DKA.

Acetone may be extracted from other bodily fluids such as in blood analysis. In a study of forty patients, researchers compared the measurements of ketones, specifically β-HB, in the blood with acetone concentrations extracted from human breath exhaled from the same human subjects [13]. Results indicate a positive correlation: that acetone concentrations increase with an increase in β-HB in the blood [13]. This indicates an association between levels of acetone extracted from human breath and β-HB, which is formed when blood glucose increases. In a separate study, researchers analyzed acetone concentration in the breath to determine whether there was an association with blood glucose concentration in type 1 diabetes during hypoglycemia [14]. Researchers applied ion flow tube mass spectrometry with eight subjects with a mean age of 28 years and a mean HbA1c level of 8.8 (glycemic control). An insulin clamp technique, a controlled stepwise reduction in plasma glucose levels, was performed over the duration of a morning [14]. Insulin clamps have been widely utilized as a metabolic tool for manipulating blood glucose levels [14]. Sample bags were used to collect breath samples from each subject. Test results indicate a positive linear correlation between plasma glucose and acetone in the breath [14].

Further research is required to determine the direct relationship between acetone and blood glucose changes during ketosis. The correlation between acetone and blood glucose raises several research questions, such as whether acetone can be adequately extracted and accurately mapped from the human breath to blood glucose levels in the presence of environmental variables, whether a sensor device can precisely support this extraction and quantify the result, and whether an associated software for decision support based on this measurement may be developed that intelligently adapts in real-time to both patient and environmental dynamic stimuli.

### 3.2. Intelligent Information Processing for Diabetic Decision Support

This section evaluates intelligent classifiers toward their integration into a dynamic system that can input the results of the blood glucose level extracted from the human breath in addition to other indicators from a patient profile, to provide decision support for diabetic lifestyle management.

Fuzzy logic relies on the principle of dynamic classification of data based on a membership function that changes its parameters according to incoming, changing data. The boundaries of classification of the data change; they are fuzzy and account for variation in data characteristics, in contrast to hard limits. Fuzzy logic uses linguistic terms embedded within membership functions for intelligent classification of dynamic data, mimicking the process of human cognitive processing. Fuzzy sets are analyzed and evaluated according to if–then rules of the fuzzy system. The development of a reliable and accurate fuzzy expert system depends on the experience of the expert [15,16]. Fuzzy logic controllers (FLCs) may prove useful for blood glucose level evaluation, given a blood glucose measurement, user input, activity selection, and other patient-specific parameters that change dynamically. Researchers have applied FLCs to classify and offer decision support for diabetic disease diagnosis [17,18,19,20], and for progression and management [18]. The FLC typically comprises a fuzzifier, which converts numeric values into fuzzy sets in the process of fuzzification, an inference engine that performs logical controls in the FLC, and a rule base, which comprises the control rules and membership functions. The results of the inference engine are then defuzzified with conversion back to numeric values.

An FLC was developed to classify a patient as diabetic or non-diabetic based on blood glucose reading input, incorporating expert knowledge with parameters including diet, medicine, and exercise [18]. This type of fuzzy expert system framework constructs an extensive knowledge base that uses defuzzification to convert fuzzy values into hard limit values. In [18], rules were constructed to implement decisions on the amount of insulin dosage required and the number of times a person would need to self-inject; affiliated semantics and corresponding quantitative correction included “increase dosage” (5%) and “decrease dosage” (9%). Results of the classifier in [18] indicate that the system effectively offered decision support to the diabetic user for self-regulation of blood glucose levels, toward the prevention of symptoms and complications.

An FLC is developed in [19] for classification of a diabetic candidate into a category denoting the stage of diabetes. Symptoms of the disease are the input parameters and fuzzy logic is applied to analyze and classify the patient into one of the four categories: type 1 diabetes, type 2 diabetes, pre-diabetes, and gestational diabetes [19]. Current patient data is entered, in addition to glucose level and historical patient data. Fuzzy logic is applied to classify this information using dynamic grading as implemented through customized membership functions [19]. While thresholds defined within the membership function may change, FLC fails to adapt its design structure based on the incoming information [19,21]. The proposed system requires adaptation in patient lifestyle for efficiency and the ability to provide precise information to the patient in critical situations. Precision is key in this area, as it can solve a situation where a patient is experiencing low blood glucose in a matter of minutes by providing the patient the required or adequate amount of glucose in order to resume proper functionality of the body during hypoglycemia.

Diabetic Decision Support Systems (DSSs) have incorporated FLCs for the analysis of diabetic blood glucose reaction to insulin infusion through the use of Mamdani fuzzy controller models [20], with the application of swarm optimization for fuzzy controller tuning. Healthy subjects were presented with minimum infused insulin and their reaction tested, against diabetics [20]. Parameter uncertainties were modeled in the FLC by degree of insensitivity to multiple meal disturbances, level of accuracy, and superiority of robustness [20]. The Mamdani fuzzy logic architecture contained two input variables and one output variable [20]. The input linguistic variables were the error signal between the measured blood glucose level and the reference glucose level, and its rate of change [20]. The output linguistic variable was the exogenous infusion rate of the insulin into the blood stream [20].

Neural networks (NNs) are composed of nodal elements and have the ability to adapt to incoming information [21]. Commonly, NNs are adjusted and trained to ensure that a specific input leads to a particular output [21]. NNs have the capability to train and process complex functions in many applications including identification, pattern recognition, speech processing, classification, and control systems [21]. NNs are implemented for diabetic diagnosis [22], for analyzing blood glucose range as a primary indicator of the diabetic condition, in addition to diabetic management and associated insulin administration [22]. NNs reveal their utility and efficiency in solving problems that require prediction, clinical diagnosis, pattern recognition, and/or image analysis. 

A system is designed in [22] to provide testing of diabetic abnormalities, where NNs are used in the early stages of diabetic diagnosis. Input parameters to the NN classifier include the number of times a patient has been pregnant, their plasma glucose concentration, blood pressure, body mass index, and insulin production level [22]. Results of the research revealed the NNs capable of learning patterns corresponding to diabetic symptoms of an individual, achieving an accuracy of 98% [22]. Prolonged, constant NN training was a noted limitation of the method, and researchers determined that, after a two-month period of no training, system accuracy dropped to 91% [22].

A hybrid approach utilizing knowledge-based expert systems, adaptive control, simulation, optimization, and NNs is also implemented for decision support for insulin administration, where the expert system mimics soft knowledge expressed by medical expert opinions, intuition, laboratory results, and clinician’s observations [22]. Parameters of interest include biological functions of blood glucose for the prediction of blood glucose response to insulin intake, coupled with decision-making and intelligent knowledge based systems combining mathematical and knowledge-based techniques [22]. The study revealed NNs to be more suitable than knowledge-based approaches in diabetic diagnosis and management applications. The options for the selection of activity for insulin administration such as age, BMI, and blood glucose levels are inflexible and exclude other important factors such as activity intensity level, dietary habits, and predictive blood glucose control [22], in addition to the inflexibility of applied uniform decision making. Experts offer non-uniform guidance and personal experience as well as patient lifestyle, which was excluded [22]. Shortcomings were identified during clinical evaluation [22] and NNs effectively addressed these deficits for more flexible lifestyle guidance with insulin administration. NNs seemingly provide a superior design structure over knowledge-based systems with the potential to enable variability of user input, patient profiling, and adaptive learning for incoming data and blood glucose-guided management for recommendations of intake and activity level based on intelligent information processing.

Decision tree (DT) approaches can be used to model a sequential decision problem under uncertainty. DTs graphically represent the decisions to be taken, events that could happen and the outcomes that may result. The structure of DTs incorporate nodes, branches, terminal values, strategy, payoff distribution, and rollback. A major objective of the system is to determine the best decision to take based on tree nodes that will constitute parametric values for consideration of a chosen path. It has previously been established that type 2 diabetes is age- and lifestyle-related, such that older persons (over the age of 40) and those that do not have healthier lifestyles are at greater risk, in comparison to younger, healthier counterparts. DTs have been utilized to identify individuals with impaired glucose metabolism or type 2 diabetes [23]. DTs have been implemented due to their clear presentation of complex data that allows easy interpretation [23].

In [23], the objective was a diabetic diagnosis through automatic generation of decision trees on a cohort of 1737 individuals without previously known diabetes [23]. Analysis included 1175 females and 562 males of the cross-sectional Metabolic Syndrome Berlin Potsdam study [23]. This test consisted of test patients of ages greater than 18 years and all without known diabetes. Exclusion criteria for the used sample in this experiment were existing diseases such as liver disease, renal failure, and/or cancer. Tested DTs included well-established risk factors of diabetes as nodes, including age, blood pressure, and fasting glucose. Results revealed that age was the greatest discriminating factor for disease diagnosis with a threshold of 48.3 years. People younger than 48.3 years were further sub-arranged by a child node based on systolic blood pressure with a limit at 127 mmHg. It was determined that the proposed DT would avoid OGTTS in about 25% of individuals with a reasonable rate of false-negative results [23]. The research determined that a sensitivity just over 30% was required to identify IFG, IGT, or T2DM [23], which does not enable the identification and classification of pre-diabetic situations [23].

Hybrid approaches utilize a combination of strategies including FLC, NNs, DTs, and/or rule-based expert systems in diabetic decision support. A hybrid system was developed that uses NN predictors with an FLC to regulate blood glucose in type 1 diabetics [24]. The NN is added into this system to avoid errors of user input that include mealtime and size. The use of NNs in future blood glucose prediction assists with the proposed control technique to handle delays connected with insulin absorption and time [24]. The FLC works alongside NNs and uses the predicted information concerning blood glucose to determine the required insulin dosage for the patient [24]. A feed forward NN and a recurrent NN are implemented and assessed as nonlinear glucose prediction models [24]. The RNN provides vastly improved expectation accuracy than the FFNN, particularly at longer prediction horizons [24]. The RNN architecture consists of three layers, two hidden layers (20 neurons and 13 neurons) and a one-output layer of one neuron [24]. The RNN is trained using the back-propagation algorithm [24], which looks for the minimum of the error function in weight space using the method of gradient descent. The FFNN architecture comprises three layers. Neurons in distinctive layers are completely joined and information or processed data is fed forward [24]. The activation function used in the two concealed layers is a sigmoid function.

The preparation and testing of the RNN and FNN prediction models are performed utilizing glucose measurements from nine subjects and patient data [25]. Most RNNs and FFNNs are tested to advance the predicted glucose values [25]. For RNNs, two hidden layers with 20 neurons and 13 neurons gave the best results [24]. In addition, observations have shown that using a predictive control strategy with a neural system as an indicator and an FLC gives glucose peaks lower than that obtained by an FLC without predictor. The proposed system was best suited for daytime usage for better regulation of postprandial glucose concentration. The RNN gives lower postprandial (period after dinner or lunch) glucose peaks than the FFNN. The FLC without a predictor does not have the ability to stay within the extreme values/threshold and thus is unable to regulate the blood glucose level. The proposed system that uses NNs and FLC to deliver the required insulin dose works well for reducing the complications of hyperglycemia by predicting the patient’s future blood glucose levels and providing decision support for insulin dosage. Within the system, four features including age, BMI, blood glucose levels, and fasting blood were used in a hybrid approach with FLC and NNs as classifiers performing weight calculation, with embedded membership functions and network logic, for diabetic diagnosis [24]. This system achieved a classification accuracy of 98.14% [24]. The method [24] may be adapted for varied classification weights, membership functions, and nodal network connections, for lifestyle management decision support including fluid recommended intake and suggested activity. Hybrid approaches that include NNs and FLCs offer greater system accuracy for diabetic management: NNs offer utility for adaptation and training of the DSS while the FLC implements the decision and classification logic [25].

## 4. Conclusions

There is an identified research deficit in the development of a system capable of non-invasive detection and intelligent classification of hypoglycemia in diabetics. In effect, an alternative method for monitoring blood glucose levels without direct extraction of a blood sample, through the examination and classification of acetone composition in the breath, is presented here. This classification is based on the measurement and intended degree of physical activity, and customized to the user, for recommendations in lifestyle management.

To address the identified research challenges, the authors propose a novel sensor-based solution capable of detecting and measuring blood glucose levels by extracting acetone from human breath, without the use of blood sampling. An algorithm is then designed and developed for processing measurements, including consideration of an embedded relationship between acetone and blood sugar levels, for real-time, dynamic, and intelligent classification. A decision support system that uses a hybrid approach combining FLCs and NNs for intelligent information processing that is patient-specific and adapts to a diabetic’s own lifestyle is presented for diabetic management in lifestyle choices, offering a customized solution that overcomes deficits in user motivation.
